# Exploration of Advanced Applications of Triboelectric Nanogenerator-Based Self-Powered Sensors in the Era of Artificial Intelligence

**DOI:** 10.3390/s25082520

**Published:** 2025-04-17

**Authors:** Yifeng Su, Dezhi Yin, Xinmao Zhao, Tong Hu, Long Liu

**Affiliations:** 1 Research & Development Institute of Northwestern Polytechnical University in Shenzhen, Shenzhen 518063, China; suyifeng@mail.nwpu.edu.cn (Y.S.); ydz0312@mail.nwpu.edu.cn (D.Y.); zhaoxinmao1002@mail.nwpu.edu.cn (X.Z.); tonghu@mail.nwpu.edu.cn (T.H.); 2 School of Electronics and Information, Northwestern Polytechnical University, Xi’an 710072, China

**Keywords:** deep learning, triboelectric nanogenerator (TENG), self-powered sensors, intelligent sensors, human–machine interaction, internet of things (IoT)

## Abstract

The integration of Deep Learning with sensor technologies has significantly advanced the field of intelligent sensing and decision making by enhancing perceptual capabilities and delivering sophisticated data analysis and processing functionalities. This review provides a comprehensive overview of the synergy between Deep Learning and sensors, with a particular focus on the applications of triboelectric nanogenerator (TENG)-based self-powered sensors combined with artificial intelligence (AI) algorithms. First, the evolution of Deep Learning is reviewed, highlighting the advantages, limitations, and application domains of several classical models. Next, the innovative applications of intelligent sensors in autonomous driving, wearable devices, and the Industrial Internet of Things (IIoT) are discussed, emphasizing the critical role of neural networks in enhancing sensor precision and intelligent processing capabilities. The review then delves into TENG-based self-powered sensors, introducing their self-powered mechanisms based on contact electrification and electrostatic induction, material selection strategies, novel structural designs, and efficient energy conversion methods. The integration of TENG-based self-powered sensors with Deep Learning algorithms is showcased through their groundbreaking applications in motion recognition, smart healthcare, smart homes, and human–machine interaction. Finally, future research directions are outlined, including multimodal data fusion, edge computing integration, and brain-inspired neuromorphic computing, to expand the application of self-powered sensors in robotics, space exploration, and other high-tech fields. This review offers theoretical and technical insights into the collaborative innovation of Deep Learning and self-powered sensor technologies, paving the way for the development of next-generation intelligent systems.

## 1. Introduction

The 2024 Nobel Prize in Physics and Chemistry has brought Deep Learning back into the global spotlight, recognizing its far-reaching impact as a cornerstone of modern artificial intelligence (AI) [1]. Deep Learning has revolutionized industrial manufacturing [2,3], biomedical research [4,5], and many other fields with its ability to mine high-dimensional features and extract meaningful information from massive amounts of data. This landmark award not only demonstrates the importance of AI as a computational paradigm but also emphasizes its enabling role in scientific discovery and technological advancement.

Artificial intelligence is broadly defined and encompasses a wide variety of algorithms. Of these, Machine Learning is often considered the dominant subset of AI algorithms, which enables machines to automatically acquire data and enhance experience as they learn, simplifying many repetitive and complex tasks in life [6]. Machine Learning is widely used in many fields such as image recognition, financial services [7], and industrial production to help people perform their work tasks more easily.

Neural networks are an important topic in the field of Machine Learning, which mimic the structure of human brain neurons to construct networks. Multilayer perceptron networks trained using “Backpropagation” algorithms, self-organizing maps, and radial basis function networks are typical examples of this technology [8,9,10]. In 2006, Hinton et al. proposed “Deep Learning” (DL) [11], which is commonly regarded as a neural network algorithm with sufficiently deep structural hierarchy and combines multiple mechanisms for extracting data features in different tasks, among which the most well-known is the Convolutional Neural Network. As an iteration and breakthrough of traditional Machine Learning algorithms, Deep Learning has shown amazing vitality in the context of significantly improved hardware technology. Deep Learning updates data and extracts features in a hierarchical manner, deepening the depth of algorithms and enabling them to tackle more complex and flexible tasks, thereby driving rapid progress in science and technology [12].

Correspondingly, sensor technology has also achieved rapid development in recent years, becoming an important tool for collecting real-world data and supporting intelligent decision making. Combined with artificial intelligence technologies, sensors not only achieve higher precision in perception, enabling the detection of weak signals and extraction of high-dimensional features, but also support edge computing, allowing for the intelligent processing of sensed data and real-time evaluation of environmental parameters. This makes sensors become an important component of the Internet of Things in the era of artificial intelligence, playing a key role in various fields from production to life. In addition, in recent years, researchers have further introduced intelligent sensors into virtual reality technology (VR), incorporating tactile perception in addition to visual perception, expanding the sensory breadth in virtual space, and making sensors a bridge connecting the real world and virtual data space [13].

Among numerous sensing technologies, the triboelectric nanogenerator (TENG) sensor stands out with its unique dual functionality, invented by Wang’s team in 2012. A TENG can directly collect energy from mechanical energy in the environment (such as vibration, wind energy, and human motion) through frictional electric effect and electrostatic induction, convert it into electrical energy, and have self-powering capabilities [14,15]. At the same time, TENG-based self-powered sensors have extremely high detection capabilities for weak mechanical signals (such as slight vibrations, pressure, or friction), with sensitivity far exceeding that of many traditional sensors, and can be used to measure various physical parameters [16,17,18]. In addition, TENG-based self-powered sensors have significant advantages, such as a simple structure, low cost, sustainability, miniaturization, and high adaptability, and have enormous potential for application in many fields. Combined with artificial intelligence technology, self-powered sensors can play an outstanding role in multimodal data fusion, complex pattern recognition, and real-time intelligent decision making. Through Machine Learning algorithms, self-powered sensors can accurately extract and analyze weak signals from the environment or human body, achieving efficient prediction, anomaly detection, and personalized response, making them widely used in fields such as intelligent healthcare [19], robot tactile perception, and Industrial Internet of Things [20,21].

Building on the aforementioned research foundation, the present review provides a comprehensive review of the applications of TENG sensors in the era of artificial intelligence. Several previous reviews have also explored the integration of AI with TENGs. For instance, Duan et al. provided an in-depth summary of the working principles and advancements of TENG sensors while discussing different categories of AI-integrated TENG systems [22]. Cao et al. and Zhou et al. examined the applications of TENGs in smart IoT and other domains [23,24], whereas Shang et al. and Tian et al. introduced the fundamental principles and operational mechanisms of Machine Learning algorithms, further analyzing their application directions based on different models [25,26]. Unlike prior reviews, the present review not only provides a holistic overview of the historical development of AI and its classic models but also systematically discusses the integration of AI with various types of sensors. Subsequently, the fundamental principles, material classifications, and operational modes of TENGs are elaborated in detail, followed by an application-oriented analysis of AI-TENG integration across different domains. In the end, the advantages [27], limitations [27,28], and challenges of this research direction are summarized, and innovative perspectives on the future development of TENG-based intelligent sensors in robotics [29,30,31], aerospace [32,33], and ocean exploration [34,35,36] are proposed.

The main contributions of this research are summarized as follows:

The present review provides a comprehensive overview of the history of Deep Learning and a performance comparison of several classical algorithms to help scholars gain a preliminary understanding of the field. It also explores various application areas and research advances in intelligent sensors. Additionally, the review introduces TENG-based self-powered sensors, discussing their principles, materials, and structures while highlighting research progress in combining self-powered sensors with artificial intelligence for applications in multiple fields. Finally, it summarizes the advantages and challenges of TENG self-powered sensors integrated with Deep Learning and proposes future development directions and potential application exploration paths [31,32,36].

Section 2 provides an overview of the history of Deep Learning development and also shows the advantages and disadvantages of several classical Machine Learning and Deep Learning approaches and their application areas, including Support Vector Machines, Long Short-Term Memory, Residual Networks, Generative Adversarial Networks, and Transformers. Section 3 provides advancements in sensor applications that combine Machine Learning and Deep Learning. Section 4 shows how the self-powered sensor works and its material advantages, as well as its various novel structures and unique efficient power conversion strategies. Section 5 shows the progress of research on self-powered sensors combined with artificial intelligence in various application areas. Section 6 outlines the future direction of intelligent self-powered sensors and summarizes the main body of the article.

## 2. Overview of Deep Learning

### 2.1. History of Deep Learning

**Figure 1:**
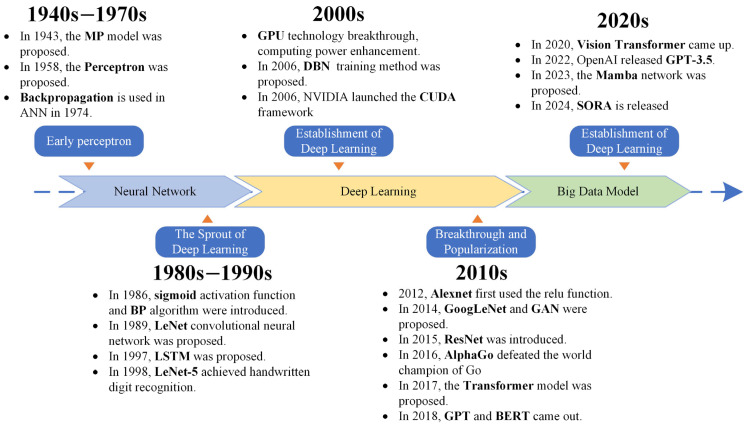
The history of Deep Learning.

As a key innovation in artificial intelligence, Deep Learning has evolved through a series of milestones over decades, as illustrated in Figure 1. Early neural network research laid the groundwork for Deep Learning’s conceptualization. For instance, the McCulloch–Pitts model (MP model), proposed in 1943, introduced a simplified neural model inspired by biological neurons, marking the earliest attempt to simulate brain-like structures [37]. In 1958, Rosenblatt proposed the perceptron [38], which could classify inputs using linear decision boundaries, an important step toward artificial neural networks (ANNs).

Subsequently, research aimed at deepening the hierarchical structure of perceptrons led to the development of multilayer perceptrons, which laid the foundation for early neural networks. The initial design inspiration for neural networks was derived from the connectivity and information processing mechanisms among neurons in the human brain; thus, they can be regarded, to a certain extent, as simplified models of human cognition. Neural networks operate on the collective functionality of a vast number of neurons, each of which is considered a processing unit or neural nucleus, equipped with multiple inputs (dendrites) and a single output (axon) [37]. Essentially, neural networks are designed to simulate the human brain’s nervous system through extensive interconnections and communications among neurons. By leveraging this biomimetic design, neural networks are capable of emulating fundamental functions in human perception, decision making, and learning, thereby replicating the operational mechanisms of the brain for predictive research and facilitating the realization of artificial intelligence technologies. However, progress was initially hindered by computational limitations and the lack of effective training algorithms.

The Backpropagation (BP) algorithm [10], introduced by Rumelhart, Hinton, and Williams in 1986, revolutionized training for multilayer neural networks. This was complemented by the introduction of the sigmoid activation function, enhancing the capacity of neural networks to handle nonlinearity. During this period, Convolutional Neural Networks (CNNs) also emerged with the introduction of convolutional layers [39]. For example, Yann LeCun’s LeNet in 1989 demonstrated significant potential in tasks like handwritten digit recognition [40], which was further refined with LeNet-5 in 1998 [41].

The late 1990s and early 2000s saw the advent of Long Short-Term Memory (LSTM), introduced by Hochreiter and Schmidhuber in 1997, tackling issues like vanishing gradients in recurrent neural networks [42,43]. Around the same time, Deep Belief Networks (DBNs), proposed by Hinton et al. in 2006, facilitated the unsupervised pre-training of deep architectures and paved the way for practical Deep Learning applications [11].

The breakthrough era of Deep Learning began in 2012, marked by AlexNet’s victory in the ImageNet competition [44]. By leveraging GPUs and ReLU activation, AlexNet achieved unprecedented accuracy in image classification tasks. This era also saw innovations such as Generative Adversarial Networks (GANs) [45,46], introduced by Ian Goodfellow in 2014, which opened new possibilities for generative tasks, and ResNet [47,48], which mitigated vanishing gradients in deeper architectures using residual connections.

Transformer models, introduced in 2017, signaled a paradigm shift by enabling powerful sequence modeling capabilities [49]. This innovation laid the groundwork for subsequent large-scale pre-trained models like BERT and GPT [50,51]. GPT-3.5, released in 2022, represented the culmination of these advances, achieving remarkable language understanding and generation abilities.

Deep Learning’s trajectory showcases the interplay of algorithmic innovation, increasing computational power, and access to massive datasets, culminating in its current widespread application across industries.

### 2.2. Comparative Analysis of Deep Learning Models

The evolution of Deep Learning has undergone remarkable changes, from the introduction of early models like the perceptron to contemporary architectures such as Transformers [6]. These developments have not only transformed the theoretical underpinnings of artificial intelligence but also broadened its practical applications across diverse domains. Building on this historical foundation, a comparison of various classical Deep Learning and Machine Learning models reveals how each addresses unique challenges while introducing specific trade-offs, as shown in Table 1.

Support Vector Machines (SVMs), as one of the earliest practical implementations of Machine Learning, gained recognition for their robust generalization ability and versatility in handling both linear and nonlinear problems, even in high-dimensional spaces [52]. However, their high computational complexity and sensitivity to missing data present significant obstacles, especially when applied to large or noisy datasets. Despite these limitations, SVMs have been widely used in text classification, image recognition, and financial risk assessment due to their simplicity and effectiveness.

In addition, K-Nearest Neighbor (KNN) and Random Forest Algorithms are also classic and excellent Machine Learning algorithms that are still widely used even nowadays. KNN is an instance-based algorithm that selects the closest K neighbors by calculating the distance of a sample point from a known sample point and predicts them based on their class. It is suitable for classification and regression tasks and is not only simple to implement but also requires no training process and is more suitable for small datasets [53]. The Random Forest Algorithm is an integrated learning method based on decision trees, which uses self-sampling and the random selection of features to construct multiple decision trees and improves prediction accuracy by voting or averaging [54]. It is resistant to overfitting, robust to high-dimensional data and missing values, and is widely used for classification, regression, and feature selection.

With the advent of Deep Learning, LSTM emerged as a solution to the challenges posed by sequential data. By effectively capturing long-distance dependencies, LSTMs have become indispensable in time-sensitive applications such as machine translation, sentiment analysis, and stock trend prediction [43]. Nonetheless, their computational complexity and reliance on large datasets often constrain their adaptability in resource-limited scenarios.

Residual Networks (ResNets), introduced to overcome gradient-related issues in deep neural networks, represent a breakthrough in architectural design. By employing residual connections, ResNet achieves high accuracy while facilitating transfer learning, making it a preferred choice for tasks such as object detection, image classification, and audio signal processing [47,48]. However, the model’s deep structure demands substantial computational resources and exhibits limited generalization capabilities on small datasets, which may lead to overfitting.

Unlike supervised learning methods, which require labeled data or target answers for model training, unsupervised learning eliminates the need for human-provided annotations. Generative Adversarial Networks (GANs) leverage unsupervised learning to produce realistic images, audio, and other forms of data, thereby revolutionizing generative modeling techniques. This algorithm operates within a zero-sum game framework by pitting two neural networks—the generator and the discriminator—against each other. The generator strives to create highly realistic samples, while the discriminator determines the authenticity of the samples by comparing generated data with real data. The entire process relies solely on the original dataset, without the need for any class labels or annotations. Their flexibility and scalability have paved the way for applications in data augmentation, anomaly detection, and privacy preservation [46]. Despite their transformative potential, GANs face challenges such as mode collapse, which can result in insufficient diversity in the generated data. Furthermore, the quality of the outputs is difficult to assess through automated metrics and often requires human supervision. Here, human supervision does not imply intervention during the training process but rather refers to the reliance on subjective human judgment during result evaluation and the manual adjustment of loss functions or training strategies when mode collapse occurs to ensure both safety and practicality.

Transformers, an innovation of the last few years, epitomize the shift towards architectures capable of capturing global dependencies with unparalleled contextual understanding. Their multi-head attention mechanism and adaptability have made them the cornerstone of modern natural language processing, computer vision, and program understanding [49,55]. However, the high computational cost of Transformers and complex hyperparameter tuning present barriers to their deployment in environments with limited resources or smaller datasets.

The evolution from traditional Machine Learning models to Deep Learning architectures epitomizes a fundamental paradigm shift—from algorithms dependent on meticulously engineered features and modest data requirements to large-scale, data-driven models that perform end-to-end learning with automatic feature extraction. Early models, such as Support Vector Machines, K-Nearest Neighbors, and Random Forests, demonstrated robust performance on small- to medium-sized datasets and excelled in interpretability; however, as data complexity and volume increased, their limitations became increasingly apparent. In contrast, the advent of Deep Learning not only broke through the bottlenecks of traditional approaches but also catalyzed revolutionary progress in fields such as computer vision and natural language processing.

Within the Deep Learning domain, model architectures have continuously evolved to meet diverse application demands. LSTM networks, by incorporating gating mechanisms, effectively capture long-range dependencies in sequential data, thereby advancing time series analysis and language modeling. ResNet addresses the vanishing gradient problem in very deep networks through the use of residual connections, enabling the learning of more complex features in a stable manner. GANs, operating within an unsupervised learning framework, have unlocked new possibilities in generating realistic images and audio despite challenges such as mode collapse. Furthermore, Transformers have transcended the limitations of traditional sequential models with their multi-head attention mechanism, successfully modeling global dependencies and establishing themselves as a cornerstone in modern natural language processing and cross-domain tasks. It is important to note, however, that the remarkable performance breakthroughs achieved through Deep Learning come at the cost of high computational complexity, training instability, and a heavy reliance on large-scale datasets.

Overall, each generation of models offers its own strengths and weaknesses, and current research trends increasingly emphasize model fusion, lightweight design, and automated tuning to adapt to evolving application scenarios and data environments. The choice of model hinges on the specific requirements of a given task, the nature of the data, and the computational infrastructure available. While traditional models like SVMs maintain relevance for simpler tasks, modern architectures such as Transformers highlight the growing complexity and capability of Machine Learning systems in addressing contemporary challenges. This interplay between innovation and application underscores the dynamic trajectory of artificial intelligence, from its foundational algorithms to the cutting-edge models shaping the future. As the unique strengths of various models become increasingly apparent, the integration of different techniques—for instance, combining traditional Machine Learning methods with Deep Learning architectures—could emerge as a widely accepted strategy [56,57]. Such a fusion not only enhances predictive accuracy while maintaining model stability but also offers breakthroughs in interpretability and robustness, all while mitigating redundant data requirements.

## 3. Sensor Applications Combined with Neural Networks

Along with the gradually powerful performance of artificial intelligence algorithms and the increasing demand for intelligence in the field of sensors, intelligent applications combining neural networks and sensors are becoming more and more common. Integrating sensors with artificial intelligence has significantly enhanced their perceptual capabilities. Leveraging the feature extraction framework of Deep Learning algorithms, these sensors can capture high-dimensional features from raw data and extract valuable information even from weak or incomplete signals. Furthermore, neural network models are capable of abstract analysis and processing of these feature data to evaluate perceptual parameters, thereby endowing sensors with intelligent data analysis functionalities. Given that the data volume generated by an individual sensor is typically limited, the deployment of lightweight network architectures on the sensor facilitates edge computing, enabling real-time and intelligent data processing.

**Figure 2:**
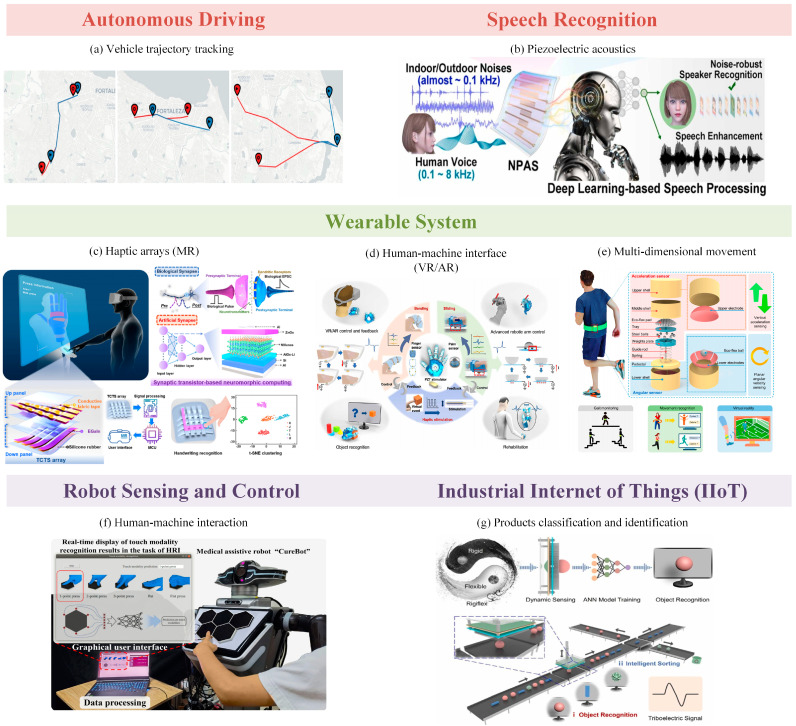
Co-design of Machine Learning and sensors. (**a**) Modeling trajectories from external sensors for location prediction using NLP (reprinted with permission [60], copyright 2024, MDPI); (**b**) Deep Learning-based noise-robust flexible piezoelectric acoustic sensors (reprinted with permission [61], copyright 2022, Elsevier); (**c**) neuromorphic computing-assisted tactile sensor array for mixed reality interaction (reprinted with permission [62], copyright 2024, American Chemical Society); (**d**) haptic-feedback smart glove as a creative Human–Machine Interface (reprinted with permission [63], copyright 2020, American Association for the Advancement of Science); (**e**) a wearable motion sensor for VR Sports (reprinted with permission [64], copyright 2023, American Association for the Advancement of Science); (**f**) bioinspired tactile sensor and Deep Learning for human–robot interaction (reprinted with permission [65], copyright 2022, John Wiley and Sons); (**g**) Machine Learning-enhanced rigiflex pillar-membrane TENG for universal stereoscopic recognition (reprinted with permission [20], copyright 2024, Elsevier).

The application of neural networks in the field of sensors gives the sensors more functions and higher flexibility. For example, Wang et al. proposed a nonlinear optical neural network for image sensing in 2023 to realize the data preprocessing of optical signals directly on the sensor to improve the sensing performance [58]. There is also Mennel and his team designing an image sensor array that acts as its own neural network, simultaneously capturing and recognizing optical images without having to convert them to a digital format [59]. Compared with traditional sensors, intelligent sensors combined with neural networks can realize high-precision information acquisition through software, provide intelligent processing and analysis functions, facilitate data sharing and remote monitoring, and carry out a certain degree of self-diagnosis and self-calibration. These features greatly enhance the sensing accuracy and breadth of the sensor, enabling it to be more widely used in various industries. Based on this zeitgeist, Figure 2 shows the combination of sensors and neural networks in various fields of application.

Autonomous driving is an extremely popular intelligent direction at present, and in the development of applications in this field, intelligent sensors show a role that cannot be ignored. Hao et al. proposed LMDrive in 2024, an instruction-following multimodal large language model (LLM) for closed-loop autonomous driving [66]. It processes camera-LIDAR data through multiple encoders and input/output adapters, enabling interaction with dynamic environments via multimodal sensor data and natural language commands. This research pioneers the use of LLMs in end-to-end autonomous driving, enhancing inference and interaction while reducing perceptual and cumulative errors. In addition, in the domain of autonomous driving, neural networks play a pivotal role in analyzing traffic conditions using sensor data. As shown in Figure 2a, Cruz et al. employed natural language processing (NLP) to encode external sensors within a road network [60], enabling the trajectory tracking and position prediction of vehicles to assess traffic conditions. Through the utilization of representation learning models in NLP, input data are transformed into a useful format that facilitates the extraction of vehicle movement trajectories and the construction of a feature space. The positioning of external sensors within the road network encodes contextual relationships in the NLP model, enabling the prediction of a vehicle’s future position based on sensor-derived trajectory data, thereby enhancing the accuracy of vehicle path tracking.

The combined application of flexible piezoelectric acoustic sensors (f-PASs) and Deep Learning is an effective component of the Artificial Intelligence Internet of Things (AIOT), where the assistance of Deep Learning equips the acoustic sensors with the ability of speech recognition and better sound enhancement. Specifically, as shown in Figure 2b, Young et al. invented a noise-robust flexible piezoelectric acoustic sensor (NPAS) by designing multiple resonant bands located outside the noise-dominant frequency range [61], which in turn achieves wide speech coverage of up to 8 khz. With the aid of a Deep Learning approach based on deep CNNs with a multi-channel attention mechanism, excellent improvements in speaker speech recognition and speech enhancement are achieved, demonstrating superior noise robustness. In addition, Reddy et al. proposed a method for recognizing hypothetical speech signals in the brain. They proposed the Multivariate Dynamic Mode Decomposition (MDMD) method and developed a framework using Random Forest and K-Nearest Neighbor algorithms for multivariate mode analysis of multi-channel electroencephalography (MC-EEG) sensor data to improve the performance of Automatic Imagined Speech Recognition (AISR) systems [67].

Additionally, smart sensing technology focusing on wearable systems is also a current research hotspot, especially for extended reality. Through integrating advanced sensor technologies with neural networks and Machine Learning algorithms, it is possible to realize real-time sensing and intelligent responses to user behavior and environmental interaction. This integration provides powerful technical support for enhancing user experience, immersion, and interactivity, and will drive personalized and intelligent extended reality applications to new heights in the future. The advent of low-cost depth-sensing technologies, exemplified by the Kinect sensor (Microsoft Corp., Redmond, WA, USA), has unlocked significant opportunities for advancing human-computer interaction applications and multimedia computing [68]. For low-cost continuous medical monitoring, Saini et al. in 2019 proposed a two-body interaction monitoring system for healthcare applications based on the Kinect sensor [69], and utilized an improved bi-directional LSTM network in conjunction with the Kinect sensor’s sensitive bone-tracking ability to track individuals suffering from mental disorders and help them recover from such disorders. Apart from this, the use of sensors to keep abreast of people’s health and make predictions about diseases is also a hot research topic in the field of wearable systems. BalaAnand and his team for this purpose proposed a wearable system connected to the IoT that collects information about a patient from the IoT and uses a Deep Learning mechanism to train the sensor data and predict the diseases, facilitating people to obtain timely and relevant therapeutic information [70].

In order to realize cross-space information communication in a Mixed Reality (MR) space using sensed multi-channel tactile data, the problem of how to obtain stable static pressure detection and dynamic execution signals on the same tactile sensor needs to be solved. Xie and his team proposed a flexible dual-mode triboelectric-capacitive coupled tactile sensor (TCTS) with an array to achieve a spatial resolution of 7 mm [62], as shown in Figure 2c. In order to expand the application areas, a single-layer perceptron (SLP)-based artificial neural network with a Backpropagation algorithm was deployed on the artificial synaptic transistor arrays to realize dynamic input signal recognition located on the sensors. In addition to this, the TCTS array system enables the visualization of static pressure intensity. This research developed a promising method for realizing the virtual reality connection, resulting in a multimedia interactive system that integrates visual and haptic senses.

The Human–Machine Interface (HMI) is an important component for realizing virtual/augmented reality (VR/AR) and plays a decisive role in enhancing the usability, ease of use, and interactive experience of the system. In 2020, Zhu et al. proposed a triboelectric-based haptic glove-type HMI (refer to Figure 2d) with knuckle-bending sensors, palm-sliding sensors, and piezoelectric mechanical stimulators [63], which achieves the detection of various degrees of freedom of the human hand and reached high accuracy object recognition using Machine Learning techniques. Through the utilization of elastomer as the sensor material and separating the finger and palm sensors, it is possible to realize multi-degree-of-freedom bending detection of the fingers while providing haptic signal feedback on the magnitude and direction of the tangential and normal forces of the palm grip. In addition, in order to further realize the function of HMI, the Machine Learning methods of SVM and CNN are used to realize object recognition, respectively. Among them, the CNN, which belongs to Deep Learning, achieves a higher recognition accuracy and also has a simpler model structure, requiring only a relatively simple neural network to achieve high performance.

In addition, wearable systems worn on other parts of the body also have rich reality enhancement features, such as sports posture monitoring. Regular exercise is an important prerequisite for a healthy life, but many exercises are usually affected by factors such as weather. To help people perform regular physical exercise indoors, researchers have proposed to assist exercise with wearable systems. As shown in Figure 2e, Guo and his team proposed a self-powered wearable multidimensional motion sensor that senses both vertical acceleration and planar angular velocity and can be integrated into a belt for gait and waist motion posture sensing [64]. The multidimensional motion sensor is categorized as an acceleration sensor and an angular sensor, enabling motion detection in any direction. Both sensors are based on the electrostatic induction effect, which utilizes triboelectricity to generate sensing signals, with different rolling friction structures and more or less output channels giving them different capabilities. Since the sensors produce near-linear voltage outputs, a simple and effective Machine Learning algorithm, SVM, is used for classification and identification, and a t-distributed stochastic neighborhood embedding (t-SNE) algorithm is used for visual clustering. The smart belt was able to recognize various movement patterns including walking, running, waist rotation, and turning with 93.8% accuracy. The team also developed a VR fitness game based on the smart belt to assist people in indoor exercise. Not coincidentally, Afsar et al. proposed a wearable sensor system for physical exercise in combination with VR games to help adolescents exercise to maintain their health [71], which uses CNNs for the feature extraction of data acquired by sensors worn on various parts of the body and RNNs for classification to achieve a high accuracy of motion detection.

Sensors play an important role as a medium for robotic systems to perceive external information. Data analysis via Deep Learning algorithms can help robots better perceive the outside world and help them interact with the outside world. In 2015, Jung et al. proposed a neural network-based gait phase classification method using sensor signals from a lower-limb exoskeleton robot as a way to accurately classify different gait phases of the robot in order to detect the user’s intent to control the robot [72]. As shown in Figure 2f, Kong et al. proposed a piezoresistive soft-touch sensor for robots and co-designed it with a bio-inspired Deep Learning-based algorithm [65]. The sensor adopts a traditional hexagonal structure, for which a novel data enhancement strategy was developed to convert the touch stimuli at different locations of the sensor into signal responses of six readout channels, which are used as input signals for the Deep Learning algorithm, significantly improving the generalization ability of the Deep Learning algorithm. A unique deep neural network (DNN) was customized for the sensor, which has some similarities with a general CNN, but in the DNN, some neuronal connections present in the CNN are eliminated to simplify the model structure and speed up convergence during training, which helps the DNN model to focus on more important information and converge faster. Based on these approaches, tactile sensors are able to detect tactile stimuli and recognize touch patterns in continuous areas during human–computer interaction, providing new insights into Deep Learning-based tactile skin for robots.

In addition to the aforementioned application directions, intelligent sensors also serve as essential components in the development of the Industrial Internet of Things (IIoT). As shown in Figure 2g, Xiong et al. introduced a rigiflex pillar-membrane triboelectric nanogenerator (rigiflex PM-TENG) enhanced with the Machine Learning technique for universal stereoscopic recognition [20], where the rigid structure senses the object’s configuration, while the flexible membrane assists in returning the rigid structure to its initial position, and the system integrates Machine Learning-based data analysis to enable real-time object recognition. In this system, self-powered sensors capture the object’s characteristic information, which is subsequently fed into a neural network model for recognition, allowing for the classification of subtle object distinctions. This system provides supplementary elements for visual recognition and paving the way for future intelligent interactions and manufacturing processes that will aid smart factories in intelligent classification and the recycling or remanufacturing of defective products. Furthermore, anomaly detection is crucial for IIoT, as industrial systems are now easily exposed to public access, making IoT devices vulnerable to attacks. Using Graph Neural Networks (GNNs) for the joint analysis of data from interconnected sensors and devices helps identify anomalies [73]. Unlike CNNs, GNNs can process non-Euclidean data, such as social network data, 3D images, and industrial data, enabling timely anomaly detection in sectors like smart transportation, energy, and manufacturing, preventing significant economic losses.

In summary, the combination of sensors and Deep Learning has driven technological innovation in multiple fields from autonomous driving and wearables to IIoT. This synergy not only enhances the functionality of sensors but also redefines the way machines perceive and interact with their environment. As Deep Learning and sensor technologies continue to evolve, the potential for their convergence will continue to lead the wave of next-generation intelligent systems and disruptive applications.

## 4. The Principle and Capacity of the Triboelectric Nanogenerator

**Figure 3:**
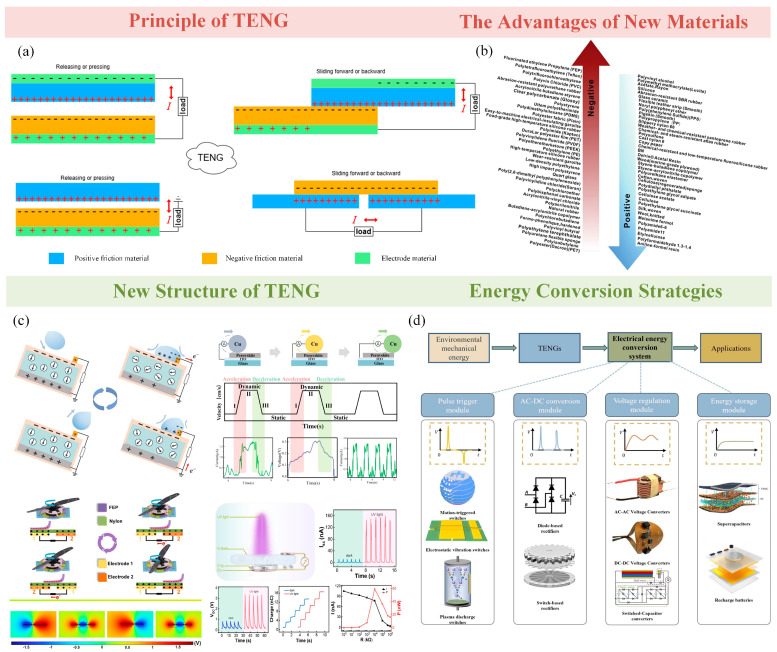
Principles and capacity of TENG. (**a**) TENG’s four modes of operation: contact separation mode, sliding mode, freestanding triboelectric layer mode, and single electrode mode; (**b**) differences in electronegativity of different materials (reprinted with permission [80], copyright 2020, John Wiley and Sons); (**c**) new TENG structure: droplet-based TENG (reprinted with permission [81], copyright 2024, Elsevier), a soft-contact TENG (reprinted with permission [82], copyright 2024, American Chemical Society), direct current TENG (reprinted with permission [83], copyright 2024, American Chemical Society), p-n junction-based TENG (reprinted with permission [84], Copyright 2021, American Chemical Society); (**d**) TENG energy conversion strategies (reprinted with permission [85], copyright 2024, Elsevier): pulse triggering, AC–DC conversion, voltage regulation, and energy storage.

With the development of modern science and technology, there is an urgent need for a technology that can harvest energy from the environment for sensing and energy supply. In 2012, Wang and his team introduced the triboelectric nanogenerator (TENG), which marked an important milestone in the field of energy harvesting [74]. The basic principle of the TENGs is based on the effects of friction electrification and electrostatic induction [75]. According to different structural designs, TENGs are mainly categorized into four common operating modes, as shown in Figure 3a, including contact separation mode [76], sliding mode [77], freestanding triboelectric layer mode [78], and single-electrode mode [79]. In contact separation mode, two materials with opposite electronegativity are contacted, and the more electronegative material tends to gain electrons and becomes a negatively charged surface. In contrast, the less electronegative material tends to lose electrons and become a positively charged surface. When the two materials are separated, the negatively charged material will cause its corresponding electrode to form a high potential, while for the positively charged material corresponding to the electrode for the low potential, the potential difference will drive the electrons from the electrode of the high potential to the electrode of the low potential, and when the two materials are in contact again, the positive and negative charges are re-neutralized, and the potential difference is reduced. At this point, the electrons in the external circuit will flow in the opposite direction, completing one cycle. In sliding mode, after the two materials are in contact when one of the surfaces slides relative to the other in the horizontal direction, the charge distribution changes, and a potential difference is generated between the electrodes, driving the current to flow in the external circuit. In the freestanding triboelectric layer mode, a free friction electric layer moves between two symmetrical electrodes. A change in the relative position between the friction layer and the electrodes results in a potential difference between the electrodes that drive current flow. In single-electrode mode, one material is in contact with and separated from another material in the ground or environment, and charge transfer occurs through coupling with a single electrode. The potential difference is generated through the single electrode as the other side is grounded or remains electrically neutral.

According to the principle of triboelectricity, the polarity of the material, i.e., the ability of the material to gain or lose electrons, has an important influence on the performance of the TENG. This polarity determines the direction of electron transfer between materials during the friction process, thus directly affecting the charge generation and output efficiency. As shown in Figure 3b, the magnitude of different materials’ electronegativity determines the tendency to gain or lose electrons during the friction process [80]. Generally speaking, the larger the electronegativity of the material, the stronger its ability to obtain electrons, and thus, it can attract more electrons and generate more negative charges. In comparison, the smaller the electronegativity of the material, the likelier it is to lose electrons, showing a stronger tendency to positive charge. Based on this property, materials with strong electronegativity are usually selected as negative materials for TENGs. Taking fluoropolymers as an example, due to the extremely strong electronegativity of the fluorine atoms in their molecules, they have a strong ability to attract electrons during friction and thus are widely used in the design and application of negative electrode materials [86]. On the other hand, materials with relatively weak electronegativity are more suitable as electron donors for positive electrode materials. For example, aminopolymers have very low electronegativity and can easily lose electrons during friction, thus becoming the main source of positive charge. However, in most practical applications, due to the poor mechanical and chemical stability of amino polymers, copper (Cu), a more stable and well-conducting metal, is usually chosen as the positive material. By rationally selecting materials with different electronegativity, a high-performance TENG system can be constructed to optimize the charge transfer and energy conversion process. The selection and combination of such materials is an important basis for the design of high-efficiency TENG devices and the key to optimizing their performance.

Meanwhile, many new materials have been invented possessing higher or lower electronegativity to capture more energy from friction. Liu et al. proposed a stress-induced adaptive phase transition strategy to conveniently fabricate self-encapsulated hydrogel-based ionically conductive fibers (se-HICFs) [87]. It possesses better contact electrostatic induction capability while ensuring mechanical strength and stability.

The new structure of the TENG is shown in Figure 3c, and the structural design of the new TENG has been constantly innovated to adapt to different application scenarios and improve the performance requirements. Yun et al. designed an innovative liquid dielectric-based TENG (LD-TENG) system with an additional liquid dielectric layer [81], as shown in Figure 3c, which utilizes the liquid as a dynamic contact medium and generates friction charge when it is contacted with and separated from the solid material, which contains a dielectric, and different dielectrics can produce different friction charges. Dielectrics and different dielectrics have different outputs in a uniform external environment as a way to distinguish different dielectrics. Luo et al. constructed a rolling-mode Cu/ternary cationic chalcogenide Schottky junction DC-TENG [83], where a copper tape is tightly wound on a customized pulley to form a positive friction layer, while the negative friction layer consists of a chalcogenide film deposited on an ITO glass, and the device efficiently guides the directional motion of frictionally excited electron-hole pairs to achieve a stable DC output. Guan et al. fabricated a soft-contact TENG [82] with FEP added to the motor, and during sliding, the FEP material and the nylon material generate negative and positive charges, respectively, and the resulting electrical signals can be used to monitor the motor speed as well as fault problems. There is also a pn-junction-based TENG [84]. Ren et al. used p-Si and n-GaN as the two friction layers, with the p-Si sliding on the n-GaN without changing the contact area and accompanied by the generation of DCs and the use of UV to increase the carrier concentration and obtain higher current and voltage output. Wang et al. designed a symmetric elastic bistable triboelectric nanogenerator (EB-TENG) [88] to collect more energy. Li et al. designed a Transverse-Asymmetric Electrode Structure TENG [89] to eliminate charge transfer losses for improving TENG output performance.

The capacitive characteristics of the TENG itself lead to its current–voltage output not being able to match that of most electronic devices. In order to solve this problem and more fully utilize the output of the TENG, researchers have developed various energy conversion strategies and dedicated circuits to improve the efficiency of the TENG. As shown in Figure 3d [85], the typical electrical energy conversion strategies are pulse triggering [90], AC-DC conversion [91], voltage regulation [92], and energy storage [93]. Among them, the pulse triggering module improves the efficiency of the initial energy harvesting. The AC-DC conversion module converts high-voltage, low-current alternating current (AC) signals into direct current (DC) outputs. The voltage regulation module ensures the stability of the output voltage, while the energy storage module can efficiently accomplish the storage and release of electrical energy according to the demand.

## 5. Triboelectric Sensors Combined with Neural Network Applications

Triboelectric sensors have attracted much attention in recent years in the fields of biosignal acquisition, motion monitoring, and human–computer interaction due to their high sensitivity, self-supplying properties, simple structure, and diverse material choices. Their ability to convert mechanical energy into electrical signals makes them outstanding in capturing weak human movements and environmental changes. Combined with a neural network algorithm based on Deep Learning, triboelectric sensors are not only capable of extracting high-dimensional features from complex nonlinear data but also of improving the recognition of behavioral patterns and environmental states through large-scale data training.

**Figure 4:**
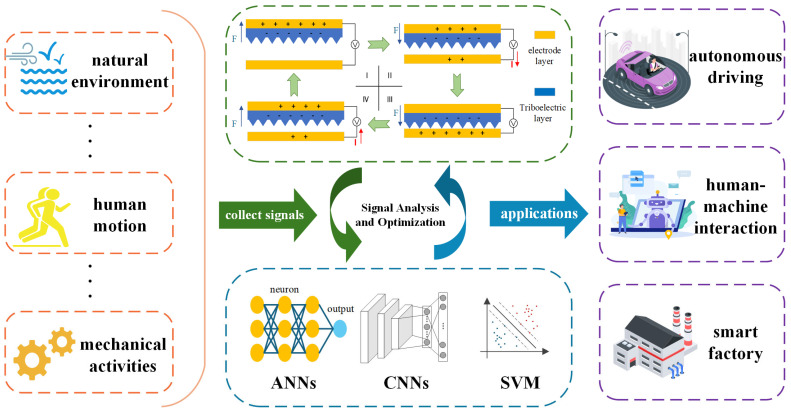
Relationship between TENG model and artificial intelligence.

These two fields—Machine Learning algorithms represented by neural networks and self-powered sensors based on TENGs—complement each other, as shown in Figure 4. Intelligent algorithms such as neural networks provide the software framework for smart sensing systems and employ self-optimization methods to dynamically adjust sensor parameters, while TENG devices continuously capture and transmit signals from the human body, environment, or machine activities. After processing by AI algorithms, these signals enable efficient recognition and decision making. This integration enhances precise perception, multimodal interaction, and real-time intelligent decision making, driving the advancement of smart sensing systems and demonstrating significant application potential in autonomous driving [94,95], human–machine interaction [96], and intelligent manufacturing [20], among others.

The integration of artificial intelligence algorithms with TENG sensors has driven the development of self-powered, highly sensitive smart sensing systems, demonstrating outstanding performance across various applications. Next, specific examples from four key areas—environmental monitoring [97,98], smart healthcare [99,100], smart homes [101,102,103], and human–computer interaction [104,105,106]—will be presented to explore their potential applications in detail.

**Figure 5:**
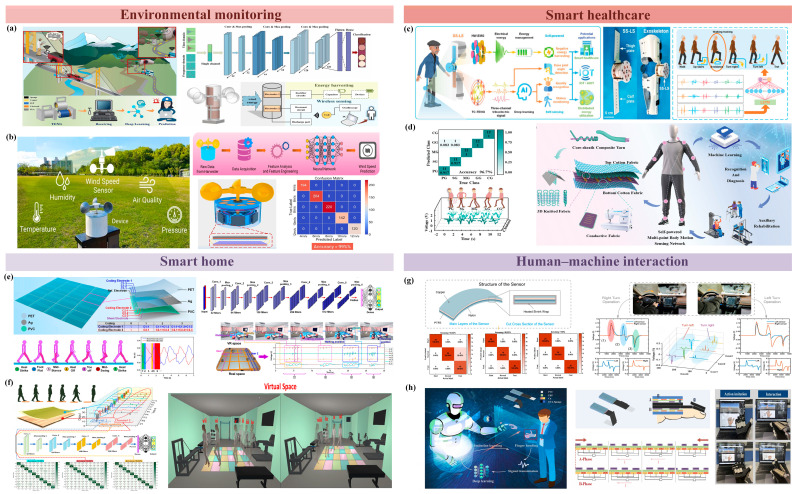
Smart triboelectric sensors. (**a**) Deep Learning-assisted self-powered wireless environmental monitoring system with multi-sensing TENGs (reprinted with permission [98], copyright 2024, Elsevier); (**b**) AI-powered TENG energy harvester for environmental monitoring (reprinted with permission [97], copyright 2022, John Wiley and Sons); (**c**) self-powered and self-sensing lower-limb system for healthcare (reprinted with permission [19], copyright 2023, John Wiley and Sons); (**d**) self-powered motion network for rehabilitation (reprinted with permission [107], copyright 2023, John Wiley and Sons); (**e**) AIoT-enabled floor monitoring for smart homes (reprinted with permission [108], copyright 2021, American Chemical Society); (**f**) robust triboelectric mat enhanced by Deep Learning for smart homes (reprinted with permission [109], copyright 2022, John Wiley and Sons); (**g**) triboelectric sensors for intelligent steering wheel in automated driving (reprinted with permission [110], copyright 2023, Elsevier); (**h**) innovative smart gloves with phalange-based triboelectric sensors as a dexterous EAI teaching interface (reprinted with permission [96], copyright 2024, Elsevier).

With the continuous development of wearable flexible sensors, the TENG has become increasingly popular due to its characteristics. In particular, the combination of TENGs and neural networks has achieved remarkable results in environmental monitoring. Liu et al. proposed a self-powered wireless environmental monitoring system based on a configurable Rotary Switch TENG (RS-TENG). As shown in Figure 5a, The rotational design of the RS-TENG enables it to function as a wind-driven device [98], efficiently harnessing wind energy through wind cups. The wind-driven RS TENG (WRS-TENG) can continuously monitor the presence of mountain debris and wind speed in real time during strong winds. The entire sensing system consists of a Machine Learning (ML) model constructed using a WRS-TENG and Convolutional Neural Network (CNN) algorithms. With a Deep Learning CNN algorithm, the relationship between the resonant frequency of the circuit and the external state can be effectively identified. In sending sample data of three types of weight data and two types of deformation data to the model, the model achieved 100% recognition accuracy after 50 rounds of learning validation. Shrestha et al. proposed a circular Halbeck array magnet with arc-shaped magnets as a high-performance rotational energy harvester for preventing magnetic flux leakage by concentrating magnetic flux in specific paths [97]. As shown in Figure 5b, the core of the system is a rotating energy harvester (HA-REH) based on a circular Halbach magnet, which prevents magnetic flux leakage by concentrating the magnetic flux on a specific path. The gap between the arc-shaped magnets of the circular Halbach array is optimized to achieve maximum magnetic flux density. The original current signal output by the TENG is captured and processed by data acquisition (DAQ). The signal through DAQ goes through the following steps: data formatting, data cleaning, and data normalization. Finally, the final data are passed to a customized artificial neural network (ANN), without considering different weather conditions.

The integration of TENG technology with neural networks has significantly advanced the field of smart healthcare. For instance, Kong et al. developed a self-powered and self-sensing lower-limb system (SS-LS) tailored for smart healthcare applications [19], as illustrated in Figure 5c. The system incorporates a three-channel triboelectric nanogenerator (TC-TENG) to precisely capture the rotation angle and direction of the knee joint, while an LSTM model is utilized to analyze the triboelectric signals. This system demonstrates exceptional performance, achieving 99.96% accuracy in classifying six distinct motion states (e.g., walking, stair climbing, turning) and detecting abnormalities such as Parkinsonian gait and falls based on real-time triboelectric data. Moreover, it provides real-time feedback by tracking joint movements during rehabilitation exercises (e.g., sit-to-stand exercises, balance training), facilitating the assessment and improvement of rehabilitation progress. Similarly, Wei et al. proposed a self-powered multipoint body motion sensing network (SMN) based on a TENG, as shown in Figure 5d [107]. This system integrates Machine Learning algorithms for biometric gait recognition and rehabilitation training. The SMN employs digital embroidery and knitting techniques to embed TENGs into textile structures, using highly flexible, stretchable, and pressure-sensitive Ag-PE core–sheath composite yarns. By combining TENG-generated signals with Machine Learning algorithms, the SMN achieved 96.7% accuracy in recognizing five pathological gaits, including Parkinson’s gait and scissors gait. Additionally, the system serves as a rehabilitation platform, enabling customized exercise guidance for patients and the tracking of their progress throughout the rehabilitation process. These innovations highlight the transformative potential of combining TENGs with advanced neural network algorithms in smart healthcare applications.

In addition to employing sensor systems to assist in medical rehabilitation training, leveraging intelligent sensor systems to empower individuals with disabilities to achieve capabilities comparable to those of non-disabled individuals represents a significant research direction. Lu and his team developed a novel lip-reading decoding system designed for individuals with speech impairments, enabling convenient and accessible communication [111]. The system features a self-powered, low-cost, flexible triboelectric sensor placed at the intersection of oral muscles, manufactured using flexible polymer films to enhance the sensing of oral skin movements. To address signal diversity and small-sample personalization challenges, a well-trained dilated recurrent neural network model based on prototype learning was implemented, achieving a test accuracy of 94.5%.

Smart homes utilize distributed electronic devices in complementary forms to achieve indoor positioning, identification, intelligent interaction, and many other functions in the home and office areas. Among them, the deployment of smart flooring in the ground area can fulfill the above functions simply and effectively while avoiding the privacy leakage problem arising from general sensing devices such as cameras. Dong and her team developed a soft, warm, and highly scalable triboelectric carpet fabric designed for motion monitoring and user identification [112]. To maintain the softness of the carpet, specially designed conductive velvet yarns were embedded within coiled threads, enabling the production of triboelectric fabric with self-powered capabilities. Furthermore, the integration of Machine Learning algorithms facilitates behavioral analysis and user identification, significantly enhancing its functional versatility. In Figure 5e, Shi et al. developed a smart floor monitoring system for a variety of smart home monitoring and interaction [108], which includes features such as trajectory tracking, identity recognition, and automatic control. The smart floor is designed based on an embedded single-electrode triboelectric sensor, and the floor is designed with an innovative electrode pattern including a reference electrode, two coded electrodes, and a sheet electrode. The large-scale sheet electrode can fully capture the human walking gait phase for identification and personalization applications, while the coding electrodes encode the different floors in the floor array for parsimonious position identification and trajectory monitoring. The ratio of the outputs of the encoding and reference electrodes is used as the sensing signal, which avoids the interference of ambient humidity and possesses relative stability. In order to facilitate feature extraction from the output time series, a one-dimensional Convolutional Neural Network (1DCNN) with multiple channel inputs is chosen to assist in the data analysis. The 1DCNN model has excellent versatility, scalability, and performance in user identification, with an average accuracy of 91.33% in recognizing user identity. In conclusion, the smart floor monitoring system obtained by integrating triboelectric coded floor mats and Deep Learning has significant advantages in large-area floor detection and has a promising future in the era of the Internet of Things (IoT).

However, the floor electrodes in the floor monitoring system designed by Shi et al. [108] only cover the middle position and the position-sensing function cannot be accurately realized in the rest of the area. Moreover, the floor mats can only track the trajectory in a one-step, one-pixel manner, and in actual walking, the user may cross any position of the floor mats and cover two mats at the same time in one step. Therefore, Yang et al. proposed a robust triboelectric carpet monitoring system equipped with rich sensing information (refer to Figure 5f) named InfoMat [109]. InfoMat contains a large-scale carpet array that consists of four groups of mats, each containing six pixels, for a total of 24 pixels (4 × 6), and the pixels in each group have different areas of the two inter-finger electrode (IDE) patterns. The output of the TENG is proportional to the area of the electrodes for the same friction area and thus has a means of distinguishing between the different pixels by taking the ratio of the outputs of the two IDEs for each pixel, making monitoring independent of environmental influences or gait. In addition, the system introduces a weight sensor with a pyramid-type structure in the carpet, which leads to higher user identification results with the assistance of a Deep Learning method based on CNN models. For InfoMat, Yang et al. [109] also designed real-time VR smart home scenarios so that the functional effects of InfoMat can be visualized and displayed in VR space and provided a method for two-way interaction between users in both real and VR space, realizing a digital twin smart home. Apart from the abovementioned design, Zhao et al. proposed a self-powered intelligent flooring system inspired by the structure of disposable paper cups [113]. The paper-cup-shaped TENG significantly increases the energy generation surface area compared to cylindrical structures, thereby enhancing its self-powered capabilities. Additionally, the flooring system integrates Deep Learning algorithms, enabling object trajectory tracking and user identification, which further expands its functional applications.

Moreover, ensuring home security is one of the important research directions in the field of smart homes. Xu et al. developed a flexible and transparent ternary-electrification-layered triboelectric membrane sensor that can adhere to curved surfaces [114]. Integrated with home devices like doors and safes, and enhanced by 1DCNN algorithms, it achieves 99.2% classification accuracy in recognizing motion states and activity patterns, enabling comprehensive smart home security monitoring.

In the field of autonomous driving, in order to improve safety and avoid traffic accidents, the driver is usually required to correct the autopilot or take over the driving of the vehicle at the necessary moments; thus, how to quickly and accurately complete the human–computer interaction between the driver and the autopilot system becomes a key issue. In 2023, Chen et al. proposed a smart steering wheel based on the triboelectric concept [110], which aimed at assisting the autopilot in order to reduce the number of traffic accidents (refer to Figure 5g). A contact-separated TENG-based sensor was integrated into the steering wheel to recognize the steering intention of the driver. A TENG-based sensor is sensitive to pressure and can detect small changes in the driver’s grip force. Self-powered sensors were placed on both sides of the steering wheel, and when the steering wheel was turned left or right, the output voltages generated by the sensors on both sides were different, thus determining the direction. Compared to the traditional steering angle sensor, the TENG-based sensor has a shorter response time and faster signal change. For the output voltage signals generated by the sensors, after data preprocessing such as filtering and differencing, the SVM-based Machine Learning method is used to classify the operating behaviors, which has good classification results. In addition, Chen et al. took advantage of the rapid response of the self-powered sensors and integrated its data into a Model Predictive Control (MCP) system to optimize the human–machine cooperative lane change control of an autonomous driving system, achieving faster steering and control response.

Embodied artificial intelligence (EAI) empowers humanoid robots to autonomously learn through intricate interactions with the external environment, including gesture-based communication, thereby serving as a highly effective tool for human–computer interaction in educational settings. As illustrated in Figure 5h, Liu et al. developed an intelligent glove incorporating triboelectric nanogenerators as an advanced teaching interface for EAI (Ti-EAI) to enhance human–robot interaction [96]. The glove integrates a phalange-based triboelectric sensor (PTS) with a segmented design that adapts to finger movements, minimizing interference from fixed structures and ensuring natural motion. The sensor employs a dual-layer electrode linkage mechanism with phase differentiation to optimize signal output and enrich gesture-related information within the signal. This is further enhanced by a Deep Learning algorithm based on multilayer convolution, enabling high-precision gesture recognition. Moreover, through integration with large language models, the intelligent glove extends beyond fundamental gesture recognition, achieving complex logical interactions and voice communication. This advancement holds immense potential for applications in human–computer interaction, facilitating more frequent and convenient communication between humanoid robots and humans, particularly in educational and gaming environments.

TENG-based touch panels have attracted much attention in the field of human–computer interaction, but how to prepare touch panels realizing large-area deformable stretchability, high-power triboelectric sensing, intelligent free-sliding recognition, and usability in extreme environments is still a current challenge, and a difficult problem. Liu et al. recently proposed a triboelectric touchpad with super stretchability and implemented Transformer-assisted intelligent gesture sliding recognition [115]. The triboelectric touchpad was prepared by stacking Ecoflex, liquid metal mesh, and antifreeze hydrogel in a cascading manner, and nine antifreeze hydrogels were placed on the surface of Ecoflex sandpaper printed with liquid metal mesh to form an array of triboelectric sensors, which resulted in a touchpad with full super-stretchability and high-pressure sensitivity. The touchpad system utilizes the Transformer algorithm in conjunction with the MLP model to parse captured sliding triboelectric signals. The ability of the Transformer algorithm to process sequential data through a self-attention mechanism makes it very effective in processing time series data, and based on the Transformer algorithm, the triboelectric touchpad system can be used to unlock a cell phone with an accuracy of 96.02%, achieving complex gesture recognition similar to that used to unlock a cell phone. In addition, the use of a triboelectric touchpad can also realize the wireless control of a drone; the sliding signal of the touchpad will be transmitted to the computer using Bluetooth and then processed by the Transformer algorithm to identify the direction of flight, and then the controller is used to control the flight of the drone. Moreover, the accuracy and timeliness of the system can still be maintained in extremely cold environments. This high-performance hydrogel triboelectric touchpad opens up new perspectives for the design of future intelligent gesture recognition remote interaction platforms with broad prospects.

Meanwhile, the combination of TENGs and neural networks has achieved remarkable results in action recognition [116,117]. Zhang et al. proposed a novel wearable triboelectric sensor array (TSA). The design is based on a flexible coplanar fork-finger electrode friction electric sensor and its array, miming the synaptic structure of neurons [117]. The sensor integrates a PVA film and an acrylic fluorescent layer through a self-assembly process to enhance the triboelectric effect. The entire sensing system consists of a TSA and a multi-input Convolutional Neural Network. Through a Deep Learning CNN algorithm, a single friction electric sensor can efficiently recognize the joint positions where movements are generated. In addition, using an improved AlexNet model, this cerebral neural network further enhances the TSA’s ability to accurately recognize a wide range of foot and gait features with 99.75% accuracy. Zheng et al. designed a stretchable, self-adhesive, and self-powered smart bandage system for motion sensing and motion intent recognition [118]. The core of the system is a bandage-type stretchable friction electric nanogenerator (BMS-TENG) with high stretchability (up to 502%), self-adhesion for easy skin fixation, and excellent breathability. The BMS-TENG utilizes the friction electric effect to convert mechanical motion into electrical signals, which enables the monitoring of human motion and physiological signals (e.g., finger bending, elbow bending, leg lifting, breathing, and swallowing). With the support of neural networks, a Machine Learning-based smart bandage system was developed to process motion signals for gesture recognition and motion intent prediction. The system monitors the toe extensor muscles of the forearm and recognizes various gestures with high accuracy (>98%) using an LSTM classifier. Furthermore, to recognizing subtle motion actions, Sun et al. proposed a Machine Learning-coupled pressure sensor array based on vertically aligned graphene triboelectric technology to detect pressure patterns generated by specific finger movements [119]. By integrating a fully connected Convolutional Neural Network, the system achieved high-precision recognition (98.1%) of sixteen different table tennis motions.

Beyond their crucial applications in the aforementioned fields, intelligent triboelectric sensors also play a vital role in industrial fault diagnosis [20,120,121,122] and intelligent sensing [123,124,125,126]. In a word, the integration of triboelectric sensors with artificial intelligence has further expanded their application potential, enabling advanced functionalities such as high-precision pattern recognition, multimodal data fusion, and real-time decision making. By leveraging Machine Learning and Deep Learning algorithms, these sensors can extract and analyze complex features from generated signals, facilitating tasks like anomaly detection, predictive maintenance, and adaptive control. This synergy not only enhances the performance and versatility of triboelectric sensors but also accelerates the development of next-generation intelligent systems in fields such as healthcare, robotics, and smart manufacturing.

## 6. Summary and Perspectives

In summary, the system combining TENG-based self-powered sensors and neural networks shows great potential for future development in the field of intelligent sensing and applications and can play a key role in the fields of smart healthcare, artificial intelligence, Internet of Things, and high-end manufacturing. Specifically, the system will be able to realize static force detection, simultaneous multi-parameter monitoring, multimodal function integration, and efficient and fast communication capabilities in the future, thus providing more accurate, efficient, and intelligent solutions in the fields of smart healthcare, smart home, and industrial automation.

Based on current research advancements, TENG-based sensors offer several advantages over conventional sensors, including self-powering capability, simple structure, low cost, high output voltage at low frequencies, and high sensitivity [27]. However, they also face significant challenges, such as rapid charge dissipation, high environmental dependency, and output signal instability [27,28]. Overcoming these limitations to ensure the stable and real-time signal output of TENG-based sensors in complex environments remains a key challenge in this field.

**Figure 6:**
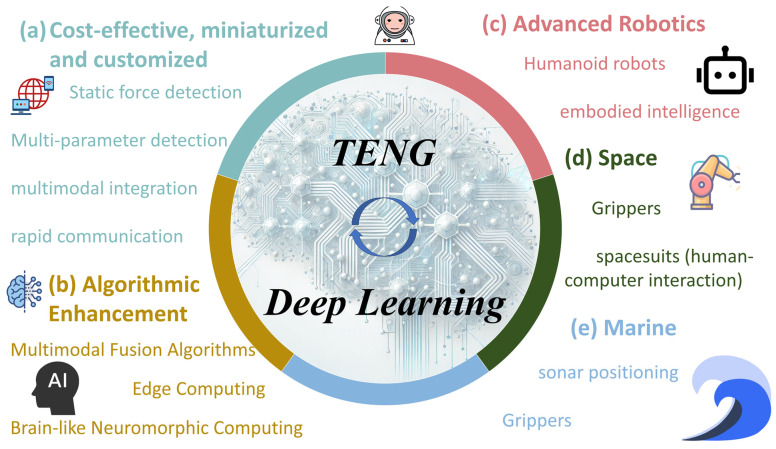
Perspectives on combination of TENG and Deep Learning. (**a**) The advantages of TENG; (**b**) the characteristics of Machine Learning algorithms; (**c**) the applications in humanoid robotics; (**d**) the applications in the field of aerospace; (**e**) the applications in the field of marine exploration.

Integrating artificial intelligence with TENG-based sensors by employing Deep Learning algorithms to predict or compensate for output signals—thereby adjusting partially attenuated or lost signals—and facilitating reliable processing and decision making based on the acquired data are a viable solution. Meanwhile, to enhance real-time performance and robustness while reducing computational costs, Deep Learning models should be designed to be lightweight for efficient edge computing. Moreover, self-optimizing algorithms can dynamically adjust parameters according to specific environmental and application requirements, and multimodal fusion algorithms can optimize the integration of multiple sensor data streams to meet diverse application demands. Consequently, achieving a high degree of coupling between TENG-based sensors and AI algorithms has emerged as an important research trend. This deep integration is expected to significantly enhance the overall performance of TENG-based intelligent sensing systems, broaden their application scope, and enable them to function effectively in increasingly complex and dynamic environments, thereby driving further innovative applications. Next, the future development potential and direction of TENG-based intelligent sensors will be explored in the three major research fields of humanoid robotics, aerospace, and ocean exploration, as illustrated in Figure 6.

In humanoid robotics, TENG-based self-powered sensors can be integrated across various components [127], such as robotic arms [128,129], biomimetic cochleae [130,131], and even to supply autonomous power [132,133]. However, these sensors often exhibit reduced stability and durability under harsh conditions such as high temperature and high humidity, which compromises their ability to accurately capture real environmental data. To address these challenges, more corrosion-resistant materials can be employed in the fabrication process, and Machine Learning algorithms can be further optimized to effectively filter out extraneous information.

Looking ahead, the integration of advanced Deep Learning models with TENG-based sensors opens several promising development directions for humanoid robotics. For instance, Deep Learning can be harnessed to develop adaptive techniques that continuously recalibrate sensor inputs in real time, thereby compensating for environmental disturbances and enhancing signal fidelity [134,135,136]. Such adaptive algorithms would allow robots to achieve high-sensitivity mechanical perception even in dynamically changing or adverse environments. Furthermore, by fusing sensing data with energy-harvesting capabilities, future humanoid robots could benefit from an intrinsic synergy between perception and power management [137,138]. Deep Learning-assisted signal processing can not only optimize data quality but also facilitate the predictive maintenance of sensor networks, ensuring long-term operational stability and reducing downtime [139]. Consequently, robots will possess enhanced capabilities to execute complex tasks, exhibiting a greater degree of embodied intelligence. Another future direction lies in the realm of human–machine interaction: TENG-based sensors integrated with AI could provide the robots with nuanced feedback regarding touch, pressure, and motion, thereby enabling more natural and efficient interactions with humans [30,31,129,140,141].

In the field of aerospace technology, TENG-based self-powered sensors can be integrated into the external operational and mobility systems of aircraft or spacecraft [32,142,143], while also serving to monitor the health status of various components of space vehicles [33,144]. However, TENG-based sensors face rigorous environmental challenges in this domain, including vacuum conditions in space and extreme temperature fluctuations, icing, gusts, thunderstorms, and low visibility within the atmosphere. For space applications, it is imperative to select materials with radiation resistance, high-temperature endurance, and corrosion resistance to cope with severe temperature gradients and intense radiation [145,146]. In contrast, for aviation applications, the structural design optimization of TENG-based sensors is essential to ensure outstanding performance under diverse and complex environmental conditions.

Machine Learning algorithms enable TENG-based sensors to dynamically adjust sensing parameters in real time, enhancing adaptability to changing environments [147]. In extreme conditions such as high temperatures, radiation, or turbulence, deep neural networks can identify anomalies and optimize sensor outputs to maintain signal accuracy and reliability. Aerospace missions necessitate multi-parameter monitoring encompassing temperature, vibration, and pressure. Due to their high sensitivity and structural flexibility, TENG-based sensors are particularly well suited for acquiring such data with precision [148,149,150]. When integrated with Machine Learning, they can localize vibration sources and detect faults, ensuring system stability. As Deep Learning and nanosensor technologies advance, edge computing is expected to be implemented on micro-scale sensor chips [151], enabling compact, low-power, high-precision monitoring, so as to meet the needs of next-generation intelligent aerospace systems.

In the field of ocean exploration, the highly dynamic and complex marine environment poses significant challenges to marine environmental sensing. TENG-based sensors offer a feasible approach for detecting the motion parameters of ocean waves and currents [152]. Owing to their self-powered nature, TENG-based sensors substantially reduce energy consumption, thereby promoting the advancement of self-sustaining marine Internet of Things applications. In tailoring the energy-harvesting structures of TENGs (such as helical, pendulum, and spherical configurations) and optimizing the contact materials, it is possible to harvest wave energy while simultaneously sensing wave-related information [153,154,155]. Although TENG-based wave sensors can provide sufficiently accurate signal outputs, they often require pre-set detection thresholds and other hyperparameters based on environmental conditions, lacking adaptability to complex marine scenarios. To address this limitation, integrating artificial intelligence algorithms can endow TENG-based sensors with dynamic self-adaptive capabilities, which represents a promising direction for future marine environmental monitoring.

Moreover, the integration of TENGs with positioning algorithms provides an effective approach for underwater information detection [156,157,158]. For instance, by collecting acoustic signals in the ocean, TENG-based sensors can be utilized for underwater sound source localization. However, there remains considerable room for improvement in terms of recognition accuracy and response speed. To address this, optimization in sensor materials, structural design, and sensor array configurations can be explored. For example, adjusting the size and thickness of the vibrating membrane or deploying multiple sensors in an array (such as a pyramid-shaped structure) can enhance data acquisition and comparison, thereby significantly improving localization precision and response time. Additionally, integrating advanced algorithmic models to optimize sonar positioning technology, combined with Deep Learning for data analysis, further enhances detection accuracy. Furthermore, the application of TENG-based underwater robotic grippers can improve the efficiency of deep-sea sampling and resource exploration [159,160]. When assisted by Deep Learning, these systems can accurately perceive the shape and hardness of target objects, enabling the adaptive adjustment of gripping force and angle [161]. Additionally, intelligent TENG-based sensors, enhanced by Deep Learning models, offer further potential in marine applications such as the underwater communication and navigation of submersible vehicles [34,35,36,162]. These applications facilitate communication and trajectory perception for divers and submersibles operating in dark and visually constrained ocean environments. However, TENG components are susceptible to corrosion in the marine environment. To mitigate this issue, highly corrosion-resistant materials or protective coatings can be employed to enhance durability and ensure sensor stability.

Therefore, the future development of the combination of TENG and Deep Learning is reflected not only in the continuous optimization of its hardware performance but also in the deep integration with advanced algorithms and multi-domain scenarios. This integration will give TENGs more powerful sensing capability, more efficient intelligent processing capability, and a wider range of application scenarios. With the continuous iteration of technology and algorithms, this combination is expected to play an important role in intelligent society construction and future technological development and become one of the core technologies promoting human scientific and technological progress.

## Figures and Tables

**Table 1:** Comparison of different Deep Learning and Machine Learning models.

Model	Advantages	Disadvantages	Applications
SVM	Good generalization and robustness	Slightly high computational complexity	Text classification, image recognition, and financial risk assessment.
	Handle linear and nonlinear problems effectively, even in high-dimensional spaces	Sensitive to missing data	
		Hyperparameter tuning is challenging	
LSTM	Effectively captures long-distance dependencies in sequences	Higher computational complexity than traditional RNNs	Machine translation, text generation, sentiment analysis, weather forecasting, stock trend prediction.
	Mitigate gradient vanishing to some extent	Require large data volumes, and limited data can weaken generalization capabilities	
	Well suited for time-sensitive data		
ResNet	Residual blocks alleviate gradient explosion and vanishing problems	Deep model structure requires significant computing resources	Object detection and segmentation, image classification, audio signal processing.
	High accuracy and suitable for transfer learning	Limited generalization on small datasets, risk of overfitting	
GAN	Capable of generating realistic images, audio, etc.	Susceptible to “mode collapse”, causing generator degradation	Image and video synthesis, anomaly detection, data augmentation, privacy encryption and protection.
	Works in an unsupervised fashion without labeled data	Challenging to evaluate generation quality, requires human intervention	
	Highly flexible and scalable		
Transformer	Multi-head attention mechanism enables parallel computation	High data requirements for effective training.	Natural language processing, computer vision, code generation, program understanding.
	Captures global dependencies with strong contextual understanding	High computational cost, complex hyperparameters, difficult tuning	
	Adaptable across multiple tasks.		
